# Supplementary material for: Analysis of Ice Phenology of Middle and Large Lakes on the Tibetan Plateau
Source: Sensors (Basel). 2023 Feb 2;23(3):1661. doi: 10.3390/s23031661 (PMC9920458; doi:10.3390/s23031661)
Supplement: Supplementary file 1 [file sensors-23-01661-s001.zip › sensors-2136083-supplementary (3).pdf]

Supplementary Materials

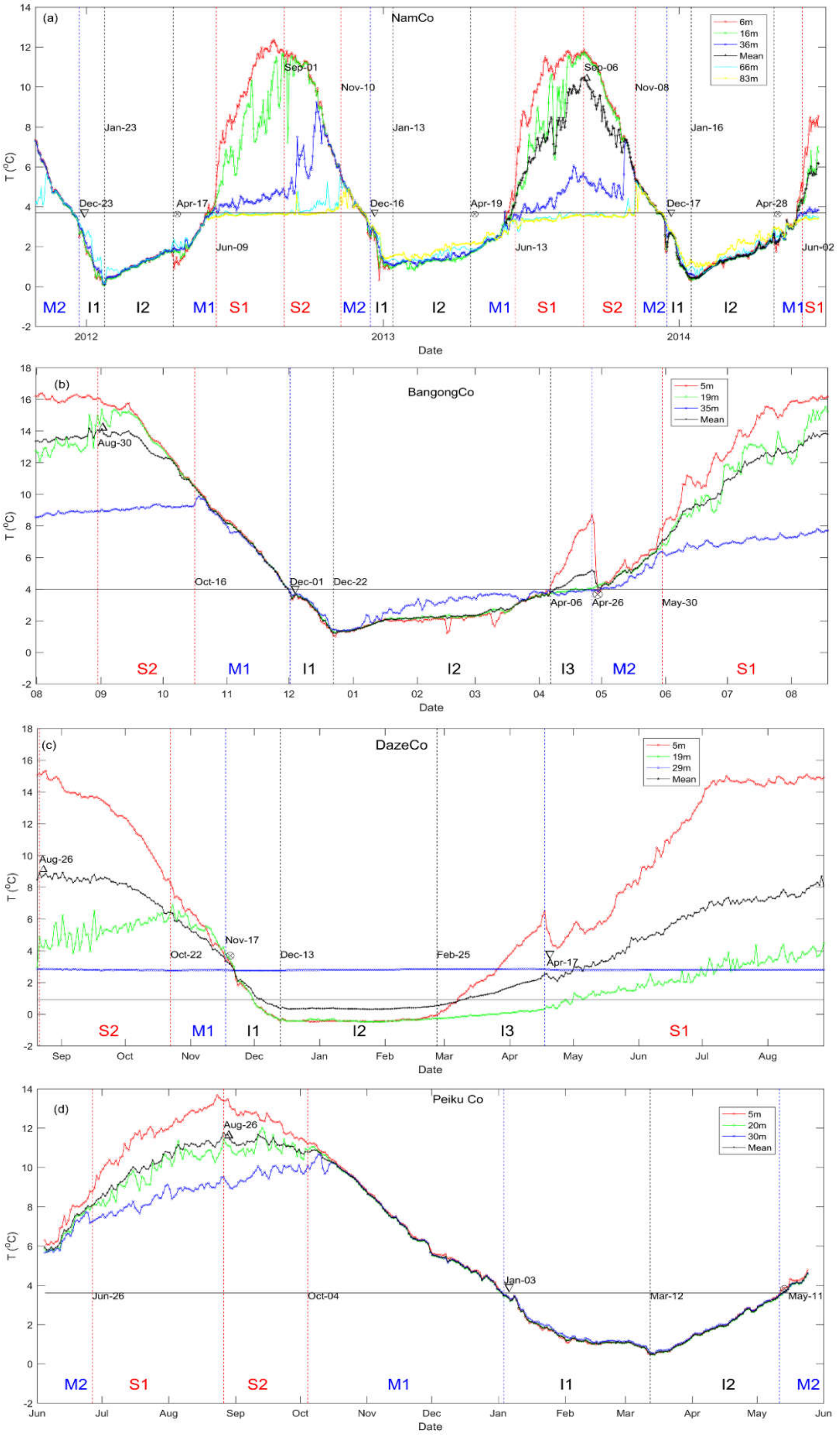

Figure S1. Lake temperature curves at different depth. (**a**) Lake temperature curves of Nam Co during November 2011-June 2014. (**b**) Lake temperature curves of Bangong Co during July 2012-August 2013. (**c**) Lake temperature curves of Daze Co during August 2012-August 2013. (**d**) Lake temperature curves of Peiku Co during June 2016-May 2017. Note: the contents will be published by Binbin Wang.

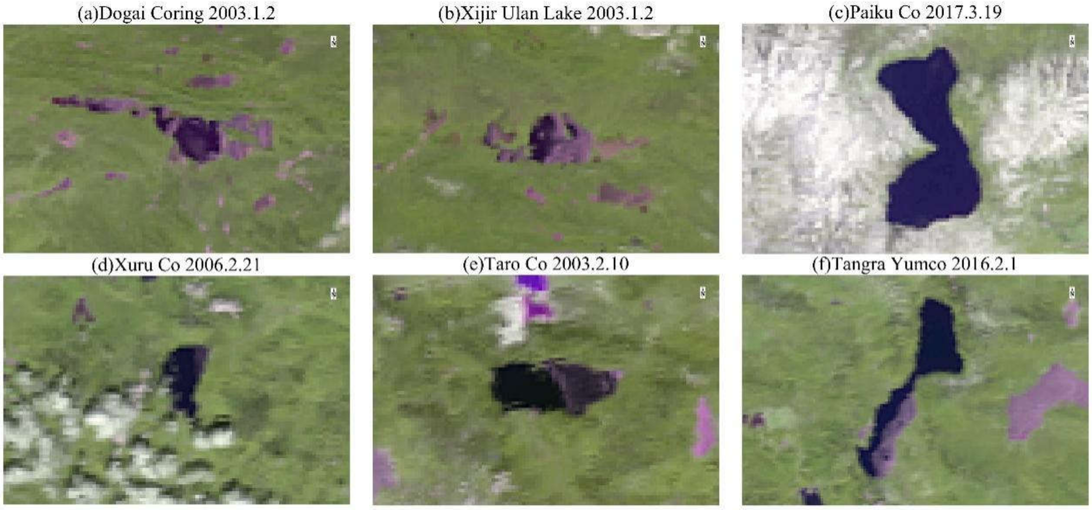

Figure S2. MODIS false-color composite image of unfrozen/incompletely frozen lakes. (**a**) MODIS false-color composite image of Dogai Coring on 2 January 2003. (**b**) MODIS false-color composite image of Xijir Ulan Lake on 2 January 2003. (**c**) MODIS false-color composite image of Paiku Co on 19 March 2017. (**d**) MODIS false-color composite image of Xuru Co on 21 February 2006. (**e**) MODIS false-color composite image of Taro Co on 10 February 2003. (**f**) MODIS false-color composite image of Tangra Yumco on 1 February 2016.

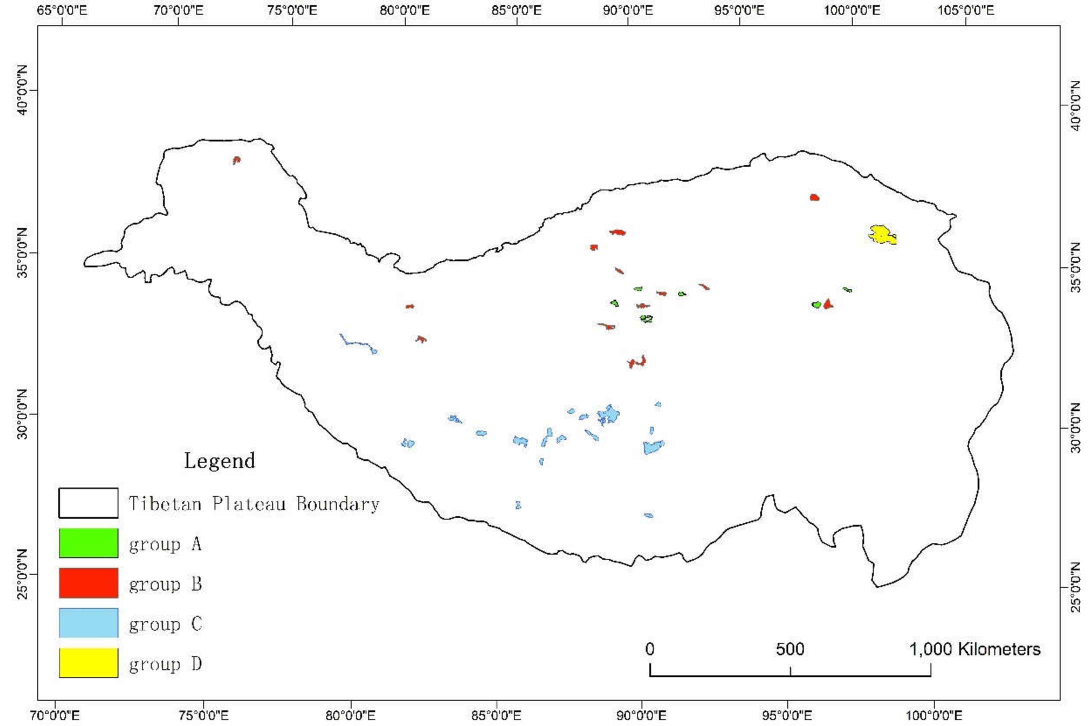

Figure S3. The classification result of lakes. Studied lakes are divided into four groups labeled as A, B, C, and D. Group A: FID9, FID13, FID14, FID15, FID18, FID19; Group B: FID1, FID2, FID3, FID4, FID6, FID7, FID8, FID10, FID11, FID12, FID16, FID17, FID20, FID40; Group C: FID5, FID22, FID23, FID24, FID25, FID26, FIID27, FID28, FID29, FID30, FID31, FID32, FID33, FID34, FID35, FID36, FID 37, FID38, FID39; Group D: FID21. FID numbers and the positions of lakes are shown in Table 1 and Figure 1.

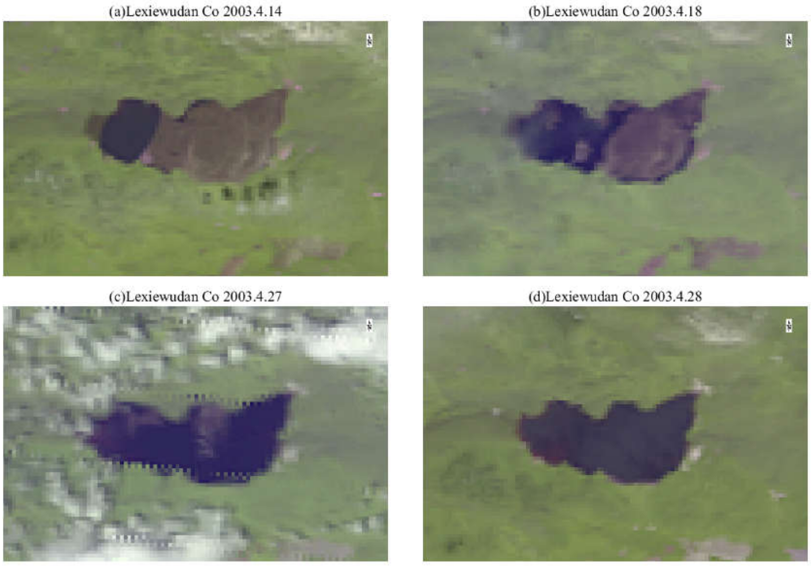

Figure S4. The ice melting process of Lexiewudan Co during 2002 to 2003. (**a**) MODIS false-color composite image of Lexiewudan Co on 14 April 2003. (**b**) MODIS false-color composite image of Lexiewudan Co on 18 April 2003. (**c**) MODIS false-color composite image of Lexiewudan Co on 27 April 2003. (**d**) MODIS false-color composite image of Lexiewudan Co on 28 April 2003.

Table S1. Unfrozen/incompletely frozen lakes during 2002 to 2021. NF is the abbreviation for not frozen, and NCF is the abbreviation for not completely frozen. Blank means frozen.

FID	Name	2002	2003	2004	2005	2006	2007	2008	2009	2010
10	Dogai Coring	NCF								
16	Xijir Ulan Lake	NF	NF	NF	NCF					
25	Taro Co	NCF								
27	Paiku Co				NF	NF		NF	NCF	
34	Tangra Yumco									
35	Xuru Co			NCF	NF	NF		NF	NF	NF
**FID**	**Name**	**2012**	**2013**	**2014**	**2015**	**2016**	**2017**	**2018**	**2019**	**2020**
10	Dogai Coring									
16	Xijir Ulan Lake									
25	Taro Co									
27	Paiku Co				NF	NF	NF			NF
34	Tangra Yumco				NCF	NCF				
35	Xuru Co	NCF	NCF		NF	NCF	NCF			NF

Table S2: Average freeze-up duration (FUD), break-up duration (BUD), lake ice cover duration (ID), and completely ice cover duration (CID) of each lake ice phenology dataset. Time between freeze-up start date and freeze-up end date determines the FUD, while time between break-up start date and break-up end date determines the BUD. ID is defined as the period between freeze-up start date and break-up end date, and CID is defined as the period between freeze-up end date and break-up start date. All variables are in "days". FID numbers indicate the lakes, with lake information shown in Table 1.

FID	Cai et al., 2019 MODIS，2001-2007				Yao et al., 2015 MODIS，2001-2011				Kropacek et al., 2013 MODIS，2001-2010				Wang et al., 2017 MODIS，2000-2015			
	FUD	BUD	ID	CID	FUD	BUD	ID	CID	FUD	BUD	ID	CID	FUD	BUD	ID	CID
1																
2									18	29	89	42	15	21	108	72
3	9	10	197	178									11	97	208	100
4			227						49	26	241	166				
5											125					
6									23	28	204	153				
7									12	23	176	141	11	23	180	146
8									16	18	212	178	14	15	213	184
9			174		17	17	179	145					13	6	185	166
10					45	51	169	73					19	19	185	147
11	15	12	180	153	19	12	188	157	28	15	200	157	20	16	196	160
12	12	10	231	209	18	13	239	208	26	29	250	195	20	16	246	210
13					14	14	213	185	30	13	228	185	36	18	217	163
14					24	48	232	160	36	36	238	166				
15			173		15	17	172	140	24	23	171	124	16	20	182	146
16					49	92	191	50	5	71	103	27				
17									47	15	194	132				
18			146										35	15	183	133
19			153										29	34	184	121
20			158										32	20	187	135
21	19	12	107	76									19	17	122	86
22									46	47	154	61				
23	9	9	106	88					24	20	116	72				
24	18	17	143	108					36	22	167	109	32	29	187	126
25			86						6	90	102	6				
26	11	13	107	83					19	19	122	84	16	17	124	91
27																

28	5	14	122	103					10	18	133	105	9	16	135	110
29			109						20	39	116	57	15	18	123	90
30									27	10	126	89				
31	17	14	120	89					30	20	142	92	15	14	121	92
32									19	18	113	76	24	27	120	69
33	7	15	119	97					13	28	133	92	14	24	135	97
34									8	71	95	16				
35									8	56	70	6				
36	6	15	136	115					9	13	146	124	12	17	152	123
37			118						34	26	132	72				
38			117						40	41	131	50	12	26	112	74
39			120						15	8	129	106	39	27	135	69
40	8	13	184	163					15	19	181	147	12	19	192	161

FID	**Guo et al., 2022** Model and MODIS, 1978-2016				**Qiu, 2018** AMSR-E, 2002-2011 AMSR-2, 2012-2016 MWRI, 2011-2016				**Qiu, 2019** passive microwave, 2002-2018, some extended to 1978				**This study** AMSR-E, 2002-2011 AMSR-2, 2012-2021			
	**FUD**	**BUD**	**ID**	**CID**	**FUD**	**BUD**	**ID**	**CID**	**FUD**	**BUD**	**ID**	**CID**	**FUD**	**BUD**	**ID**	**CID**
1													18	16	182	148
2	18	5	101	78					16	13	84	55	9	10	117	98
3	12	9	198	177	8	10	201	183	6	22	205	177	8	13	204	183
4	22	13	226	191					12	18	230	200	6	9	221	206
5	26	29	140	85					7	6	103	90	7	6	105	92
6	12	16	187	159	8	14	184	162	11	12	177	154	8	11	181	162
7	11	12	166	143	8	11	178	159	9	11	172	152	11	14	182	157
8	17	22	223	184									6	11	211	194
9	12	4	174	158	7	11	189	171					6	10	195	179
10					13	10	110	87	25	20	103	58	14	15	115	86
11	21	13	183	149	9	8	181	164	6	10	183	167	6	10	183	167
12	18	15	236	203	11	13	233	209	16	24	238	198	9	13	235	213
13	16	13	215	186	8	9	207	190					7	14	207	186
14	28	48	223	147	17	21	223	185					16	13	221	192
15	13	15	180	152	13	15	182	154	7	8	166	151	11	13	194	170
16	27	25	165	113					12	12	142	118	17	14	150	119
17	49	19	207	139					8	9	178	161	8	10	181	163
18	18	12	154	124					8	12	152	132	8	13	158	137
19	31	29	175	115	25	21	167	121	14	16	159	129	6	11	156	139
20	28	10	167	129	21	14	161	126	9	11	159	139	11	14	165	140
21	19	11	109	79	10	9	103	84	18	22	121	81	18	14	116	84
22	32	14	154	108	11	14	136	111	16	15	128	97	5	10	130	115
23	11	14	106	81	14	12	115	89					10	10	106	86
24	28	16	157	113	14	10	145	121	11	14	142	117	13	9	147	125
25	7	76	98	15									9	11	82	62

26	14	14	113	85	8	8	106	90	12	13	108	83	14	13	115	88
27	27	21	92	44									10	11	85	64
28	8	16	125	101	7	7	126	112	9	19	115	87	9	8	123	106
29	21	68	121	32									7	9	105	89
30	47	66	151	38					8	12	136	116	8	13	120	99
31	16	11	121	94	8	10	110	92	18	20	125	87	14	12	115	89
32	25	19	113	69									5	10	108	93
33	11	30	125	84	9	12	125	104					6	9	124	109
34													10	11	89	68
35													15	16	88	57
36	7	16	139	116	7	7	139	125					10	8	136	118
37	18	24	128	86	12	7	123	104					10	9	123	104
38	22	39	130	69	8	15	102	79	17	28	113	68	11	11	96	74
39	11	41	129	77									10	10	126	106
40	17	27	189	145	12	13	190	165	11	16	182	155	8	12	192	172

Table S3. Average values of basic characteristics and ice phenology for each lake group. The number of lakes in each group (N), altitude (Alt), latitude (Lat), lake area (Area), average temperature in December (T-12), average temperature in June (T-6), annual shortwave radiation (SW), and snow depth (SD) are considered. Lake ice phenology parameters include freeze-up start date (FUS), freeze-up end date (FUE), break-up start date (BUS), break-up end date (BUE), and the period between freeze-up start date and break-up end date (ID).

Group	N	Alt (m)	Lat (°)	Area (km^2)	T-12 (K)	T-6 (K)	SW (Wm^{-2})	SD (m)
A	6	4608.8	35.3	339.9	250.8	270.6	237.4	0.09
B	14	4560.5	35.8	412.3	252.8	270.7	239.7	0.01
C	19	4601.7	31.1	589.8	260.6	275.6	254.6	0.01
D	1	3191	37	4204.5	261.3	280.3	219.5	0

Group	FUS	FUE	BUS	BUE	ID (day)
A	11-Nov	28-Nov	30-Apr	18-May	188
B	19-Nov	6-Dec	1-May	20-May	182
C	24-Dec	8-Jan	29-Mar	21-Apr	118
D	14-Dec	31-Dec	23-Mar	6-Apr	113

Table S4. Lake ice phenology parameters during 2002 to 2021 in Nam Co extracted by the western pixel.

Year	FUS	FUE	BUS	BUE
2002-2003	2003/1/13	2003/1/20	2003/4/5	2003/4/26
2003-2004	2004/1/16	2004/1/26	2004/3/25	2004/4/12
2004-2005	2005/1/16	2005/2/2	2005/4/27	2005/5/8
2005-2006	2006/1/24	2006/1/31	2006/4/9	2006/4/23
2006-2007	2007/1/22	2007/2/1	2007/4/23	2007/5/3
2007-2008	2008/1/21	2008/2/1	2008/4/28	2008/5/16
2008-2009	NCF	NCF	NCF	NCF
2009-2010	2010/1/25	2010/2/6	2010/3/30	2010/4/11
2010-2011	2011/1/26	2011/2/3	2011/4/26	2011/5/11
2012-2013	2013/1/20	2013/1/23	2013/5/9	2013/5/19
2013-2014	2014/1/14	2014/1/21	2014/4/17	2014/4/29
2014-2015	2015/1/8	2015/1/17	2015/4/9	2015/4/27
2015-2016	NCF	NCF	NCF	NCF
2016-2017	NCF	NCF	NCF	NCF
2017-2018	2018/2/14	2018/2/22	2018/4/15	2018/5/3
2018-2019	2019/1/7	2019/1/16	2019/4/23	2019/5/6
2019-2020	2020/1/19	2020/1/27	2020/4/27	2020/5/12
2020-2021	2021/2/9	2021/2/23	2021/3/28	2021/4/11

Table S5. Lake ice phenology parameters during 2002 to 2021 in Nam Co extracted by the eastern pixel.

Year	FUS	FUE	BUS	BUE
2002-2003	2003/1/14	2003/1/18	2003/4/19	2003/5/4
2003-2004	2004/1/8	2004/1/26	2004/4/14	2004/4/29
2004-2005	2005/1/19	2005/1/30	2005/4/25	2005/5/9
2005-2006	2006/1/22	2006/1/28	2006/4/14	2006/4/26
2006-2007	2007/1/18	2007/1/31	2007/4/28	2007/5/8
2007-2008	2008/1/21	2008/1/30	2008/4/26	2008/5/10
2008-2009	2009/2/1	2009/2/6	2009/4/12	2009/4/27
2009-2010	2010/1/19	2010/2/8	2010/4/6	2010/4/21
2010-2011	2011/1/27	2011/2/2	2011/5/2	2011/5/10
2012-2013	2013/1/2	2013/1/10	2013/5/9	2013/5/24
2013-2014	2014/1/12	2014/1/13	2014/4/29	2014/5/11
2014-2015	2015/1/1	2015/1/12	2015/4/20	2015/4/28
2015-2016	2016/1/20	2016/1/31	2016/4/2	2016/4/14
2016-2017	2017/2/3	2017/2/18	2017/4/18	2017/4/26
2017-2018	2018/2/8	2018/2/12	2018/4/19	2018/5/6
2018-2019	2019/1/3	2019/1/12	2019/4/22	2019/5/2
2019-2020	2020/1/20	2020/1/29	2020/5/4	2020/5/11
2020-2021	2021/2/9	2021/2/16	2021/4/11	2021/4/21

Description of extraction method of lake ice phenological events:

The following example will be used to describe the extraction process of lake ice phenological parameters. Only one pixel in the center of the lake is selected to judge the freezing-thawing dates according to the change of brightness temperature. The linear interpolation method is used to complete the missing brightness temperature data, and the median filtering method is used to denoise the brightness temperature sequence. On this basis, Formula 1-6 was used to extract the phenological parameters of lake ice, and finally visual interpretation was used to correct the extraction results. The specific extraction process is as follows (The determination of lake ice phenological parameters in Nam Co during 2005-2006 was taken as an example):

(1) The brightness temperature sequence after interpolation and filtering is denoted as TB, and the brightness temperature on the ith day is denoted as TB_i (i=1,2,3... ,365 or 366). Combined with TB, a 7-day moving time window was selected to calculate the time series of brightness temperature difference, which was denoted as DTB. The brightness temperature difference on the i-3 day can be calculated by:

$DTB_{i-3}=(TB_i+TB_{i-1}+TB_{i-2}+TB_{i-3})/4-(TB_i+TB_{i+1}+TB_{i+2}+TB_{i+3})/4$, i=4,5,6…,362 or 363

For example:

TB sequence is: TB_1, TB_2, TB_3…, TB_{359}, TB_{360}, TB_{361}, TB_{362}, TB_{363}, TB_{364}, TB_{365}

DTB sequence is: DTB_1, DTB_2, DTB_3…, DTB_i…, DTB_{362}

where

$DTB_1=(TB_1+TB_2+TB_3+TB_4)/4-(TB_4+TB_5+TB_6+TB_7)/4$

$DTB_2=(TB_2+TB_3+TB_4+TB_5)/4-(TB_5+TB_6+TB_7+TB_8)/4$

…

$DTB_{i-3}=(TB_i+TB_{i-1}+TB_{i-2}+TB_{i-3})/4-(TB_i+TB_{i+1}+TB_{i+2}+TB_{i+3})/4$

…

$DTB_{362}=(TB_{359}+TB_{360}+TB_{361}+TB_{362})/4-(TB_{362}+TB_{363}+TB_{364}+TB_{365})/4$

(2) Since the brightness temperature changes most obviously during freeze-up and break-up, the brightness temperature curve will present an elevated platform, and the edge of the elevated platform corresponds to FUE and BUS (Figure S5). The brightness temperature difference time series (DTB) can reflect the speed of brightness temperature increase/decrease, so the minimum and maximum values of DTB can be calculated to determine the approximate dates of freeze-up end and break-up start (denoted as FUE1, BUS1), which respectively correspond to equations 1 and 2. The specific dates of freeze-up end and break-up start are determined by FUE1, BUS1 and the brightness temperature threshold. The brightness temperature threshold is obtained by calculating the mean value of the brightness temperature near FUE1 and BUS1 in a month. Within 10 days near FUE1 (or BUS1), find the dates whose brightness temperature are greater than the brightness temperature threshold, and among them, select the date with the minimum (or maximum) brightness temperature difference as the final FUE (or BUS). The flow chart for determining FUE is as follow, where the time step is introduced to make the brightness temperature threshold as large as possible, so as to ensure that FUE and BUS are at the edge of brightness temperature platform.The results are shown in Figure S5.

$$FUE1 = \min((TB_i+TB_{i-1}+TB_{i-2}+TB_{i-3})/4-(TB_i+TB_{i+1}+TB_{i+2}+TB_{i+3})/4) = \min (DTB_{i-3}), \quad (S1)$$

$$BUS1 = \max((TB_i+TB_{i-1}+TB_{i-2}+TB_{i-3})/4-(TB_i+TB_{i+1}+TB_{i+2}+TB_{i+3})/4) = \max (DTB_{i-3}), \quad (S2)$$

where i=4,5,6…,362 or 363

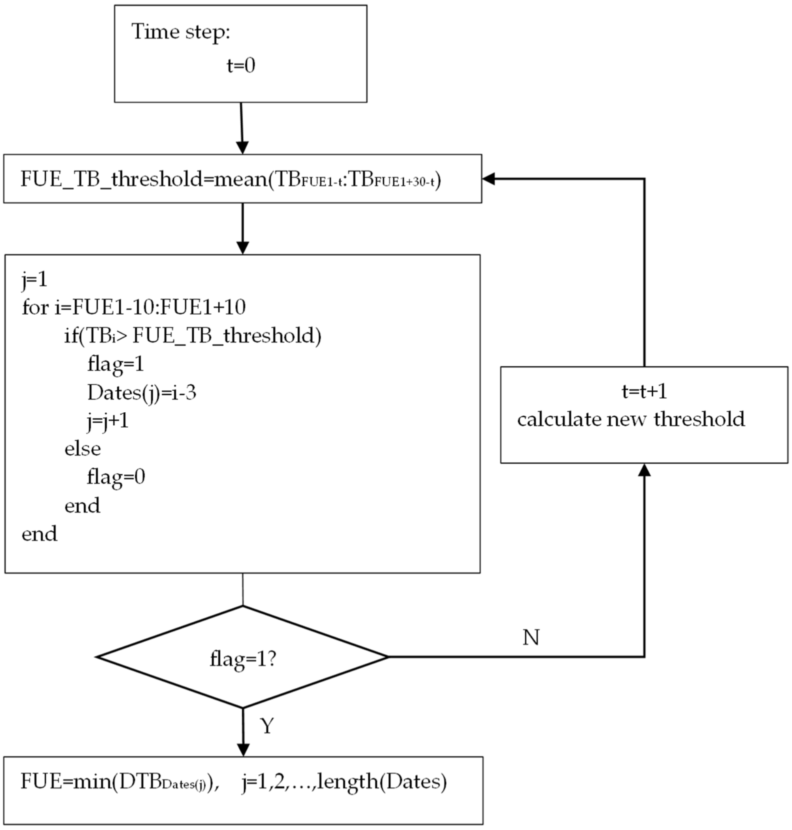

(3) The positive and negative curve intersection method of Qiu et al.(Qiu et al., 2018) combined with the brightness temperature threshold was used to determine FUS and BUE. The specific operation is as follows: Find the intersection points of Y1 (Equation (3)) and Y4 (Equation (6)) curves, and select the third closest intersection point on the left side of FUE as FUS1; Find the intersection points of Y2 (Equation (4)) and Y3 (Equation (5)) curves, and select the third closest intersection point on the right side of BUS as BUE1; The average brightness temperature of a month before FUS1 is used as the threshold of FUS, and the first minimum brightness temperature point less than the threshold on the left of FUE is selected as the final FUS; The average brightness temperature of a month after BUE1 is used as the threshold of BUE, and the first minimum brightness temperature point less than the threshold on the right of BUS is selected as the final BUE. (Figure S5)

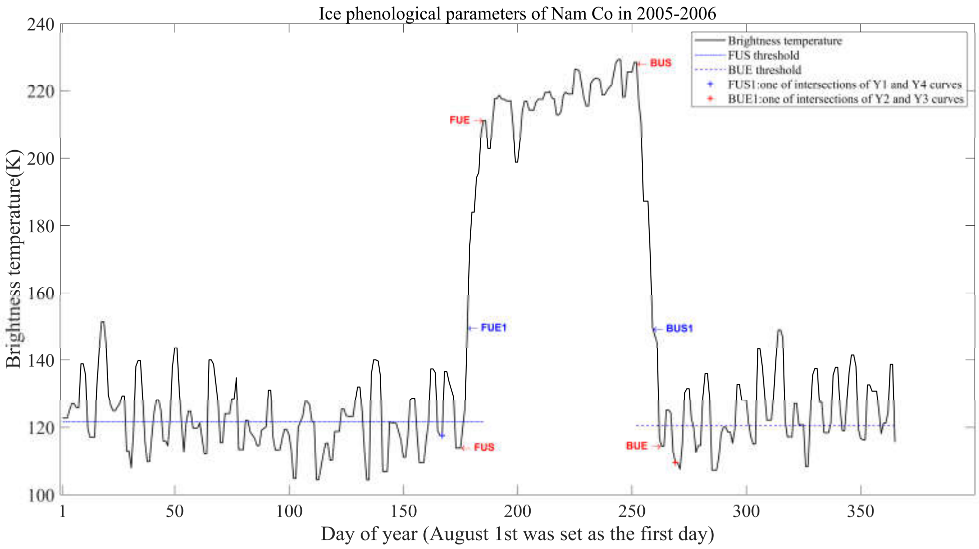

Figure S5. Determination of phenological parameters of Nam Co in 2005–2006.

(4) Finally, the visual interpretation results of brightness temperature curves were used to verify the automatic extraction results of lake ice phenological dates. If extraction results are not reasonable, visual interpretation should be used instead.

References:

Qiu, Y.; Wang, X.; Ruan, Y.; Xie, P, Passive microwave remote sensing of lake freeze-thawing over Qinghai-Tibet Plateau. *J. Lake Sci.* **2018**, *30*, 1438–1449.
